# Seroprevalence of Toscana virus in dogs from Corsica, France

**DOI:** 10.1186/s13071-016-1665-4

**Published:** 2016-07-01

**Authors:** Mustapha Dahmani, Sulaf Alwassouf, Sébastien Grech-Angelini, Jean-Lou Marié, Bernard Davoust, Rémi N. Charrel

**Affiliations:** Research Unit of Emerging Infectious and Tropical Diseases (URMITE) UMR CNRS 7278 IRD 198 INSERM U1015, Aix-Marseille University, Marseille, France; UMR “Emergence des Pathologies Virales” (EPV: Aix-Marseille University - IRD 190 - Inserm 1207 - EHESP), Marseille, France; Fondation Méditerranée Infection, APHM Public Hospitals of Marseille, Marseille, France; French National Institute for Agricultural Research (INRA), LRDE UR045, Corte, France; Working Group of Animal Epidemiology of French Army Health Service, DRSSA Toulon, French Military Health Service Academy – École du Val-de-Grâce, Paris, France

**Keywords:** Toscana virus, Dog, Corsica, France, Sand fly, *Phlebotomus*, Meningitis

## Abstract

**Background:**

Toscana virus (TOSV) is an arbovirus belonging to the Bunyaviridae, a family of negative-stranded, enveloped RNA viruses. The virus can be transmitted to humans through the bite of an infected female sand fly of the genus *Phlebotomus*. Infections are usually asymptomatic but the virus is known to cause aseptic meningitis and/or meningo-encephalitis in the Mediterranean countries. Dogs are good sentinels for detection of viral circulation and are more easily accessible than wild animals.

**Findings:**

In 2013 and 2014, we collected sera from 231 adult dogs living in 26 counties in two departments in Corsica, a French island in the Mediterranean. The virus microneutralization-based seroprevalence assay revealed a seropositivity of 3.9 % dogs on the eastern coast of Corsica.

**Conclusions:**

Our study confirms the circulation of TOSV in Corsica. Accordingly, in geographical areas where dogs possess TOSV neutralizing antibodies, direct and indirect TOSV diagnosis should be implemented in patients presenting with febrile illnesses and central nervous system infections such as meningitis and encephalitis.

## Background

Toscana virus (TOSV) is an arbovirus belonging to the Bunyaviridae, a family of negative-stranded, enveloped RNA viruses. The virus can be transmitted to humans through the bite of an infected female sand fly of the genus *Phlebotomus*. The infection has previously been reported in countries located on the northern shores of the Mediterranean Sea (Italy, Croatia, France, Greece, Portugal and Spain), as well as in the east (Cyprus and Turkey) [1] and, recently, from North Africa (Morocco, Tunisia and Algeria) [2, 3]. Although it is believed that asymptomatic infections are frequent, TOSV is an important cause of aseptic meningitis and meningo-encephalitis, during the warm season (April to October) when sand flies are active [1]. In France, the first case of Toscana virus infection was reported in a German tourist returning from the region of Marseille, south-eastern France [4]. Since then, in France several autochthonous cases of TOSV infection have been described causing either meningitis [5, 6] or encephalitis [7]. Furthermore, myositis was reported as an additional clinical complication of TOSV virus [8].

In Corsica, the seroprevalence among human blood donors was 8.7 % in 2007 [9]. The presence of TOSV in *P. perniciosus* sand flies was revealed using PCR targeting the L-RNA segment [10]. Genetic analysis showed that Corsican TOSV belongs to lineage A [10]. Dogs and humans live in close contact and are both bitten by sand flies [11], the main transmission vectors of dog and human leishmaniasis. In Corsica, dogs are exposed to the bites of sand flies as evidenced by the rate of incidence of canine leishmaniasis [12]. Consequently, the aim of our study was to evaluate the TOSV seroprevalence in dogs in Corsica, a French island in the Mediterranean.

## Methods

**Fig. 1:**
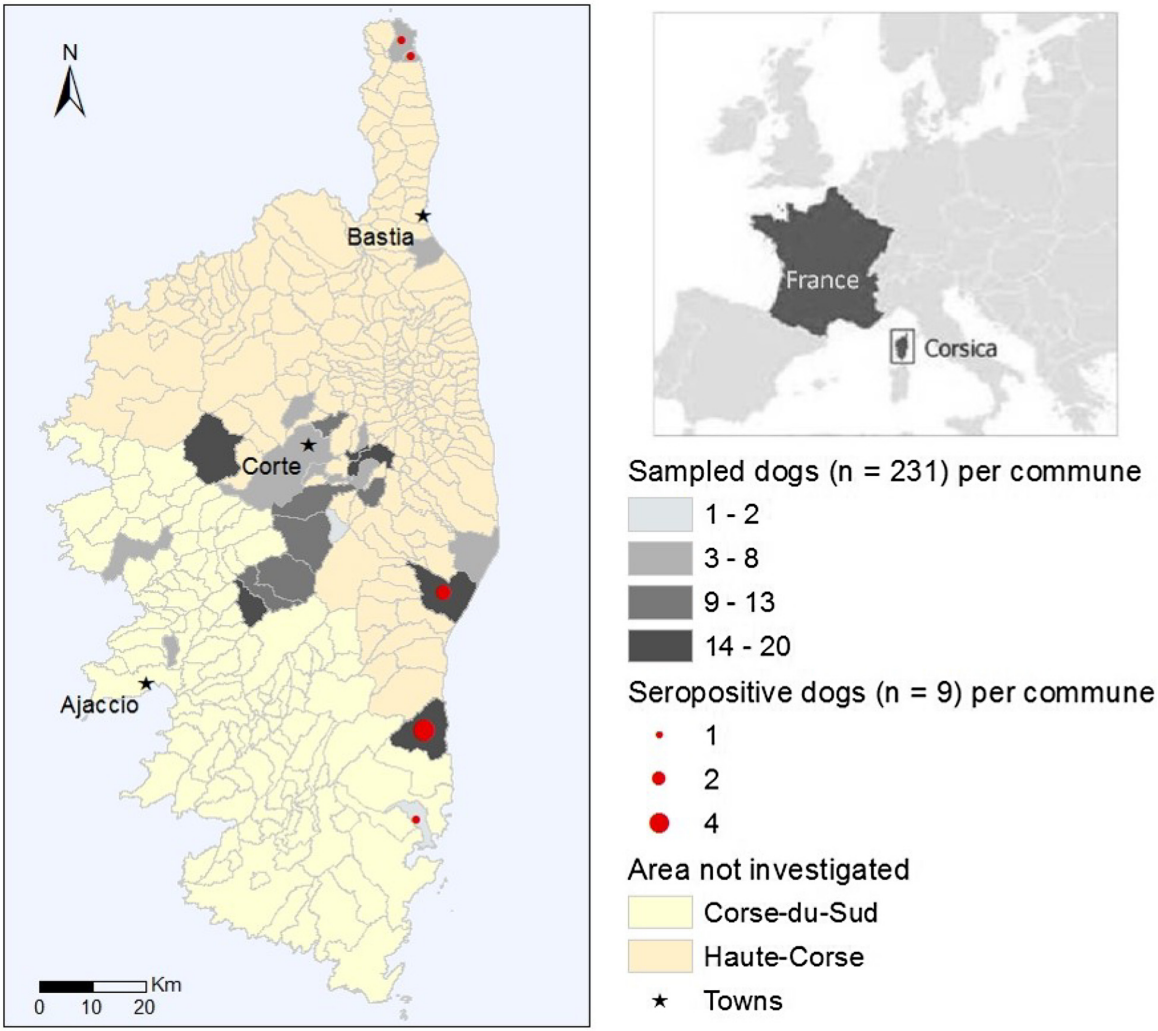
Map of Corsica showing the areas where dogs were sampled and where positive dogs were found

In 2013 and 2014, we collected sera from the radial veins of 231 adult dogs. The dogs were living in 26 communes in the two departments of Corsica (Fig. 1): in Haute-Corse [Aleria (n = 8), Biguglia (*n* = 4), Castirla (*n* = 7), Corte (*n* = 8), Erbajolo (*n* = 7), Favalello (*n* = 17), Ghisonaccia (*n* = 16), Mauracciole (*n* = 2), Riventosa (*n* = 4), Rogliano (*n* = 3), San’Andréa-di-Bozio (*n* = 15), Sermano (*n* = 4), Tavera (*n* = 12), Tomino (*n* = 4), Tralonca (*n* = 9), Ucciani (*n* = 15), Venaco (*n* = 11), Ventiseri (*n* = 20), Vico (*n* = 6) and Vivario (*n* = 9)] and in Corse-du-Sud [Afa (*n* = 7), Albertacce (*n* = 17), Altiani (*n* = 13), Brognano (*n* = 9), Casanova (*n* = 3) and Lecci (*n* = 1)]. Among the 231 dogs sampled, 147 were male (63.6 %), and 84 were female (36.4 %). The average age of dogs was four year-old (3–12 year-old) and most were wearing collars to reduce the likelihood of arthropod infestation. The dogs came from a range of sources including hunters’ dogs, shepherds’ dogs, military working dogs and some pet dogs. They all appeared to be in good health at the time of sampling and were examined with the assistance of their owners. Blood samples were centrifuged within 24 h of collection and the sera were subsequently frozen at -20 °C until being processed in the laboratory.

The seroprevalence study based on virus microneutralization (MN) was adapted from the previously described protocol [3]. The MN assay was performed in 96-well microtitre plates using Vero cells. In short, two-fold serial dilutions of 50 μl-serum aliquots were mixed with an equal volume of 1,000 TCID50 (Tissue Culture Infective Dose producing pathological change in 50 % of the cell culture inoculated) of TOSV (strain MRS2010-4319501) into 96-well plates, providing two-fold final dilutions between 1:20 and 1:160. Controls consisted of each serum (1:10) with Vero cells but without virus. After five days, the microplates were analysed under an inverted microscope, and the presence (neutralization titre at 10, 20, 40, 80 and 160) or absence (no neutralization) of cytopathic effect was noted. Cut-off titre for positivity was 20 [3, 13].

## Results and discussion

The virus microneutralization-based assay is the most discriminative serological test adapted to differentiate the affinity of antibodies against different closely related viruses [14]. Of the 231 dogs, nine (eight males and one female) were seropositive (3.9 %) for TOSV antibodies. The seropositive dogs were all from five communes in the Haute-Corse [Tomino 1 positive/4 tested, Rogliano 1/3, Ghisonaccia 2/16, Ventiseri 4/20] and one from the Corse-du-Sud [Lecci 1/1] (Table 1). Seropositive dogs were all from the eastern coast of Corsica. This region has an average altitude of 37 m, while all the dogs from the central region were negative, corresponding to a region with a medium altitude of 576 m. The positive dogs were aged between three and 12 year-old (Table 1). The serological titres were low: 1/20 (*n* = 5) and 1/40 (*n* = 4), which was expected when a virus challenge dose of 1000TCID50 was used, thus rendering the assay more stringent compared with the commonly used 100TCID50; previous studies have shown a good correlation between both protocols [3]. The results from two TOSV neutralization-based seroprevalence studies reported in Tunisia were comparable with the results of the present study. In Kairouan, 5.6 % (11/147) of the seropositive dogs were living in an area with a high density of sand flies and where leishmaniasis is endemic [2]. In Bizerte, where leishmaniasis cases are uncommon, none of the 118 tested dogs was TOSV seropositive [2]. In Algeria (Kabylia), one serological survey showed 4.3 % positive sera among 93 dogs [15]. In contrast, the results reported from Turkey were much higher. The TOSV MN seroprevalence reported in southern Anatolia was 40.4 % (21/52); of these dogs, 15.5 % (24/155) were TOSV viraemic. In addition, two dogs were co-infected with *Leishmania infantum* [16]. In Granada (Spain), 48.3 % (138/286) of the dogs tested were positive for TOSV using indirect immunofluorescence (IFAT) [17]. However, this result should be considered with caution due to the possibility of cross-reactivity with other phleboviruses such as Granada virus, Massilia virus, Naples virus or Tehran virus [3]. This is the reason why we decided to use neutralization assay which is the most discriminative serological technique [18] and which is not hampered by cross-reactions due to antibodies elicited by sand fly-borne phleboviruses either from other antigenic groups (Sandfly fever Sicilian and Salehabad viruses) or from related viruses belonging to the Sandfly fever Naples species distinct from TOSV.

**Table 1:** Location, age and serological titres (microneutralization) of Toscana virus seropositive dogs

Departement	Commune	Localisation	Age of dog	Titer
Haute-Corse	Tomino	42°56'44"N, 9°26'28"E	12	40
	Rogliano	42°57'25"N, 9°25'08"E	6	20
	Ghisonaccia	42°00'59''N, 9°24'18''E	4	20
			9	40
	Ventiseri	41°55'36''N, 9°24'19''E	3	20
			9	20
			9	40
			9	20
Corse du Sud	Lecci	41°40'48"N, 9°19'05"E	10	40

The seroprevalence of TOSV found in dogs in this study was fairly low. Historical records describe *P. mascitii*, *P. perniciosus* and *Sergentomyia minuta* on the whole island at altitudes lower than 800 m, with the latter two sand fly species being largely dominant. *Phlebotomus mascitii* has been described only in the north-eastern region. *Phlebotomus papatasi, P. ariasi* and *P. perfiliewi* were never reported [19]. Thus, the absence of seropositive dogs from the centre of Corsica is probably best explained by the fact that sand flies are rare in this mountainous region that seems to be colder than the flat coastal region. Furthermore, the use of insecticide collars for the prevention of leishmaniasis has probably reduced the number of infected dogs by decreasing the sand fly bites [20]. Our results suggest that the question of whether dogs serve as a reservoir for the Toscana virus remains unanswered and merits further environmental and experimental studies. Despite the lack of TOSV serological studies using neutralization tests in Corsican human populations, the results presented here, together with the direct evidence of TOSV in sand flies and high ELISA-based seroprevalence in blood donors [21], suggest that dogs are good sentinels for serosurvey studies of the type discussed. Our study contributes to the identification of Corsican areas where the virus currently circulates, and shows that the east coast is subject to substantial viral circulation. Accordingly, geographic areas where dogs possess TOSV neutralizing antibodies are good candidates for implementing direct and indirect TOSV diagnosis in patients presenting with febrile illnesses and central nervous system infections such as meningitis and encephalitis.

## Conclusions

Our study indicates that dogs are promising sentinels for exposure to TOSV transmission by sand flies in the Mediterranean region. However, the potential of dogs as reservoirs of Toscana virus is yet unknown. Our data extend the knowledge of the distribution of this virus in Corsica, indicating higher prevalence of Toscana virus along the eastern coasts of Corsica. In geographic areas where dogs possess TOSV neutralizing antibodies, direct and indirect TOSV diagnosis should be implemented in patients presenting with febrile illnesses and central nervous system infections such as meningitis and encephalitis.

## Abbreviations

ELISA, enzyme-linked immunosorbent assay; MN, microneutralization; TCID50, tissue culture infective dose producing pathological change in 50 % of the cell culture inoculated; TOSV, Toscana virus
